# The endosymbiont *Wolbachia* increases insulin/IGF-like signalling in *Drosophila*

**DOI:** 10.1098/rspb.2009.0778

**Published:** 2009-08-19

**Authors:** Tomoatsu Ikeya, Susan Broughton, Nazif Alic, Richard Grandison, Linda Partridge

**Affiliations:** UCL Institute of Healthy Ageing and GEE, University College London, Darwin Building, Gower Street, London WC1E 6BT, UK

**Keywords:** *Drosophila*, insulin-like signalling, *Wolbachia*

## Abstract

Insulin/IGF-like signalling (IIS) is an evolutionarily conserved pathway that has diverse functions in multi-cellular organisms. Mutations that reduce IIS can have pleiotropic effects on growth, development, metabolic homeostasis, fecundity, stress resistance and lifespan. IIS is also modified by extrinsic factors. For instance, in the fruitfly *Drosophila melanogaster*, both nutrition and stress can alter the activity of the pathway. Here, we test experimentally the hypothesis that a widespread endosymbiont of arthropods, *Wolbachia pipientis*, can alter the degree to which mutations in genes encoding IIS components affect IIS and its resultant phenotypes. *Wolbachia* infection, which is widespread in *D. melanogaster* in nature and has been estimated to infect 30 per cent of strains in the Bloomington stock centre, can affect broad aspects of insect physiology, particularly traits associated with reproduction. We measured a range of IIS-related phenotypes in flies ubiquitously mutant for IIS in the presence and absence of *Wolbachia*. We show that removal of *Wolbachia* further reduces IIS and hence enhances the mutant phenotypes, suggesting that *Wolbachia* normally acts to increase insulin signalling. This effect of *Wolbachia* infection on IIS could have an evolutionary explanation, and has some implications for studies of IIS in *Drosophila* and other organisms that harbour endosymbionts.

## 1. Introduction

The insulin/IGF-like signalling (IIS) pathway is ubiquitous in multi-cellular animals and may have been involved in the evolution of multi-cellularity itself (Skorokhod *et al*. 1999). The pathway, via a conserved intracellular signalling cascade including phosphatidylinositol-3-kinase (PI3K) and protein kinase B (PKB or AKT), negatively regulates the activity of several transcription factors of the forkhead family, by their phosphorylation and subsequent exclusion from the nucleus. Mutations that reduce the activity of the IIS signalling cascade have pleiotropic effects on many traits, including growth (Brogiolo *et al*. 2001; Butler & Le Roith 2001; Ikeya *et al*. 2002; Rulifson *et al*. 2002), development (Kimura *et al*. 1997), metabolic homeostasis (Saltiel & Kahn 2001), adult lifespan, resistance to stress (Lithgow *et al*. 1995; Clancy *et al*. 2001; Holzenberger *et al*. 2003) and fecundity (Partridge & Gems 2002; Liang *et al*. 2003; Nelson & Padgett 2003; Tatar *et al*. 2003; Broughton *et al*. 2005; Piper *et al*. 2008) in the nematode worm *Caenrhabditis elegans*, the fruitfly *Drosophila melanogaster* and the mouse *Mus musculus*. Natural variation in some ecologically significant traits has also been found to be associated with IIS. For example, allelic variants of the PI3K are implicated in the latitudinal variation of reproductive diapause in *Drosophila melanogaster* (Williams *et al*. 2006).

A primary function of IIS is to match energetically demanding activities such as growth and reproduction to nutrition and other informative environmental inputs such as various stresses, and to ensure metabolic homeostasis. Thus, consistent with its role in nutrient and stress sensing, a variety of extrinsic factors can be important regulators of insulin sensitivity in animals and humans. For example, the stress-responsive Jun-N-terminal kinase (JNK) pathway regulates IIS, linking responses to environmental stresses with metabolic homeostasis (see Karpac & Jasper 2009 for review). In both *C. elegans* and *Drosophila*, genetically upregulating JNK signalling leads to decreased IIS, increased resistance to oxidative stress and extension of lifespan (Oh *et al*. 2005; Wang *et al*. 2005). The evolutionarily conserved nutrient-sensing TOR pathway regulates protein synthesis and growth in response to amino acids and growth factors (Wullschleger *et al*. 2006), and interacts with IIS to control lifespan in worms and flies (Vellai *et al*. 2003; Jia *et al*. 2004; Kapahi *et al*. 2004). In humans, diet (Romao & Roth 2008), stress (Depke *et al*. 2008) and exercise (Colberg 2007) have all been shown to affect insulin sensitivity. Acute (transient) insensitivity can also be experienced following infections and injuries in humans (Ikezu *et al*. 1997; Del Aguila *et al*. 2000; Van den Berghe *et al*. 2001), and, in *Drosophila*, infection by pathogenic *Mycobacterium* has been found to alter IIS (Dionne *et al*. 2006).

Another possible infectious extrinsic factor modifying IIS in *Drosophila* is *Wolbachia pipientis*, a micro-organism of the rickettsial family. *Wolbachia* is a widespread endosymbiont of arthropods and often causes infection-induced reproductive incompatibility in the host. This *Wolbachia*-induced trait has been found to occur in many *Drosophila* species, including *D. melanogaster* (Bourtzis *et al*. 1996; Veneti *et al*. 2003; McGraw & O'Neill 2004). *Wolbachia* infection can also affect other aspects of host reproduction, and feminization, male killing and parthenogenesis have all been reported (Stouthamer *et al*. 1999; Hurst *et al*. 2000). Although the relationship between *Wolbachia* and the host species is usually symbiotic, a virulent form of *Wolbachia* that shortens lifespan has been found in *D. melanogaster* (Min & Benzer 1997). In the Bloomington stock centre, 30 per cent of *D. melanogaster* strains have been estimated to be infected by endosymbionts (Clark *et al*. 2005). Removal of *Wolbachia* by tetracycline treatment showed that it was involved in the sterility of the *Sxl*^*4*^ mutant allele (Starr & Cline 2002) and the lifespan-extension phenotype of an *Indy* mutant stock (Toivonen *et al*. 2007). The mechanisms involved in these effects of *Wolbachia* are not yet understood.

In the course of specifying and standardizing the *Wolbachia* infection status of our *D. melanogaster* laboratory stocks, we observed that the removal of *Wolbachia* by tetracycline appeared to exacerbate IIS mutant phenotypes. We therefore made a systematic investigation by measuring the effect of *Wolbachia* infection status on a range of IIS-related phenotypes in control and IIS mutant flies. In the presence of *Wolbachia*, ubiquitous expression of a dominant negative form of the insulin receptor (InRDN) resulted in moderate dwarfism, reduced fecundity and extension of female lifespan, all typical phenotypes of reduced IIS. In the absence of *Wolbachia*, the previously moderate effects of InRDN expression were accentuated, resulting in flies that showed severe IIS-related phenotypes including extreme dwarfism, sterility, increased fat levels and shortened lifespan. The absence of *Wolbachia* in control genotypes resulted in a decrease in fecundity and adult weight compared with infected control genotypes, but no effect on other IIS-related phenotypes. These data suggest that *Wolbachia* can increase IIS in infected flies.

## 2. Material and methods

### (a) Fly stocks and husbandry

yw;UAS-dInR^A1409K^ (chr. II) was obtained from the Bloomington *Drosophila* Stock Centre (BDSC; ref. number. FBal0156359). The UAS-InRDN transgene causes an amino acid substitution in the kinase domain (R1409A) of the *Drosophila* insulin receptor (dInR), resulting in its dominant negative activity (Wu *et al*. 2005). w^1118^; Daughterless-GAL4 (chr. III) (Fly Base ID FBti0013991) was obtained from the BDSC. UAS-PTEN was provided by Prof. Ernst Hafen (ETH, Switzerland), and w^1118^;;S_1_,106-GeneSwitch (chr. III) is described in Giannakou *et al*. (2004). The *white*^Dahomey^ (*w*^Dah^) background stock is described in Broughton *et al*. (2005). The parental GAL4 and UAS strains used to generate experimental and control genotypes were backcrossed to our laboratory control strain *w*^Dah^ (*Wolbachia*-infected) to standardize genetic background, beginning with an initial cross between *w*^Dah^ females and transgenic males, followed by five subsequent backcrosses between transgenic females and *w*^Dah^ males. All stocks were maintained and experiments performed on standard sugar/yeast food as described in Bass *et al*. (2007), except food containing 160 g l^−1^ yeast was used for GeneSwitch experiments using Mifepristone (RU486, Sigma).

### (b) Generation of *Wolbachia*-infected and -uninfected flies

**Figure 1.**
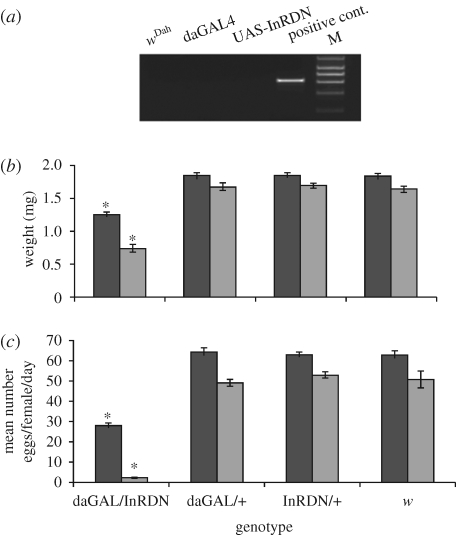
The effect of *Wolbachia* infection status on weight and fecundity of IIS mutant and control female flies. (*a*) PCR to the *wsp* gene demonstrating the removal of *Wolbachia* from parental strains. Positive control was genomic DNA obtained from tetracycline-untreated *w*^Dah^ strain. (*b*) The body weight of 7-day-old adult females was examined (*n* = 12): Wol+ w^Dah^ = 1.819 mg; Wol− *w*^Dah^ = 1.628 mg; Wol+ InRDN/+ = 1.829 mg; Wol− InRDN/+ = 1.683 mg; Wol+ daGAL4/dInRDN = 1.239 mg; Wol− daGAL4/dInRDN = 0.727 mg; Wol+ daGAL4/+ = 1.83 mg; and Wol− daGAL4/+ = 1.66 mg. Black bars, *Wolbachia*-infected; grey bars, -uninfected. (*c*) Average number of eggs laid per female per day at two weeks of age (*n* = 8 vials of 10 flies): Wol+ *w*^Dah^ = 62.76 eggs; Wol− *w*^Dah^ = 50.65 eggs; Wol+ InRDN/+ = 62.72 eggs; Wol− InRDN/+ = 52.53 eggs; Wol+ daGAL4/InRDN = 27.7 eggs; Wol− daGAL4/InRDN = 1.98 eggs; Wol+ daGAL/+ = 63.73 eggs; and Wol− daGAL/+ = 49.1 eggs. (*b*,*c*) Weight and fecundity data were subjected to two-way ANOVAs. Genotype (*p* < 0.0001) and *Wolbachia* status (*p* < 0.0001) had significant effects on both phenotypes, and there was a significant interaction effect on weight (*p* = 0.0238) and fecundity (*p* < 0.0001). Planned comparisons of weight and fecundity by genotype in Wol+ or Wol− backgrounds were performed using the Tukey HSD, *p* < 0.05. Error bars are s.e.m. and asterisk indicates significant difference from genetic controls. The statistical analyses of planned comparisons of weight and fecundity by *Wolbachia* status for each genotype are shown in table 1.

PCR to detect the *wsp* gene (Zhou *et al*. 1998) confirmed that, after backcrossing, all stocks were infected with *Wolbachia* (Wol+; data not shown). To create genetically identical *Wolbachia*-uninfected stocks (Wol−), the backcrossed Wol+ GAL4, UAS and *w*^Dah^ strains were treated with 30 µg ml^−1^ of tetracycline contained in standard food for two generations and retested by PCR to confirm that *Wolbachia* had been removed (figure 1*a*). All stocks were then cultured under normal conditions for at least five generations to allow full recovery from the effects of tetracycline treatment and to restore intestinal flora (Holden *et al*. 1993; Toivonen *et al*. 2007) prior to the generation of *Wolbachia*-infected and -uninfected daGAL4/UAS-InRDN, daGAL4/+, UAS-InRDN/+ and *w*^Dah^ genotypes for further analysis.

### (c) Lifespan experiments

Procedures for lifespan studies were as described in Clancy *et al*. (2001) and Mair *et al*. (2003). The experimental flies were allowed to mate for 48 h before collection and sorting. Lifespans were measured in flies kept at 10 per vial on standard food medium and transferred to new food three times a week. Deaths were scored five to six times in every 7 days.

### (d) Fecundity and body weight

Females for measuring body weight were prepared as for lifespan experiments and weighed individually when 7 days old. For fecundity, the number of eggs laid per vial of 10 females per day in lifespan experiments was counted.

### (e) Triacylglycerol assay

Triacylglyceride (TAG) content quantification was performed as described in Grönke *et al*. (2003), except that data were normalized to whole fly weight.

### (f) Oil red O staining

Lipid droplets were stained with Oil Red O (Sigma; Gutierrez *et al*. 2007). Seven-day-old (adult) female abdomens were attached to a glass slide using double-sided tape and dissected to expose the adult fat body attached to the dorsal abdomen. Samples were embedded in Vectashield with DAPI (Vector). Images were taken using an ORKA-ER CCD camera (Hamamatsu Photonics) and an Axioskop 2 microscope (Zeiss), and subsequently analysed using ImageJ.

### (g) AKT and FOXO protein detection

Anti-phospho-Drosophila Akt (no. 4054) and Akt (no. 9272) antibodies were purchased from New England BioLab (UK distributor for The Cell Signalling). Anti-dFOXO is described in Giannakou *et al*. (2007). Five flies were homogenized with 200 µl of 1× sample buffer containing proteinase inhibitor and phosphatase inhibitor cocktail I (Sigma). SDS–PAGE analysis was performed using a BioRad mini gel system and the proteins were transferred to a membrane by semidry blotter. Primary AKT antibodies were used at 1∶1000 dilution and the dFOXO primary antibody at a dilution of 1∶000. A secondary antibody conjugated with HRP was used at 1∶10 000, and the signal developed using an ECL detection kit (GE Healthcare).

### (h) Statistical analyses

Statistical analyses were performed using JMP (v. 7) software (SAS Institute). Lifespan data were subjected to log-rank tests and are presented as survival curves. Weight, fecundity, TAG levels and fat-cell number data were tested for normality using the Shapiro–Wilk *W*-test on studentized residuals (Sokal & Rohlf 1998). Two-way (genotype, *Wolbachia* status) analyses of variance (ANOVA) were performed and planned comparisons of means were made using the Tukey HSD test or Student's *t*, as appropriate. Data are presented as means of raw values ± s.e.m., and asterisk denotes significant difference from controls.

## 3. Results

### (a) Removal of *Wolbachia* enhances IGF-like signalling mutant phenotypes

To determine how *Wolbachia* infection status affects IIS, we characterized typical IIS-related phenotypes in *Wolbachia*-infected and -uninfected IIS mutant flies and their controls in parallel. IIS mutant flies were created by expression of a dominant negative form of the insulin receptor (UAS-InRDN) under the control of the ubiquitous and constitutive daughterless-GAL4 (daGAL4) driver.

Reduced growth and fecundity are typical phenotypes of lowered IIS and, as expected, daGAL4/InRDN females weighed significantly less and laid fewer eggs than their daGAL/+, InRDN/+ and *w* control genotypes, irrespective of *Wolbachia* status (figure 1*b*,*c*; and electronic supplementary material, figure 1*a*–*d*). However, removal of *Wolbachia* greatly accentuated the reduction in weight and fecundity of the daGAL4/InRDN genotype and moderately reduced these phenotypes in control genotypes (figure 1*b*,*c*; and table 1 for statistical analysis by *Wolbachia* status).

**Table 1.** Statistical comparisons of mean weight and fecundity data from figure 1*b*,*c* by *Wolbachia* status. Two-way ANOVAs were performed, which found genotype and *Wolbachia* status to be the main effects. Planned comparisons of mean weight and fecundity of *Wolbachia*-infected (Wol+) and -uninfected (Wol−) flies for each genotype were performed using the Tukey HSD.

genotype	weight			fecundity		
	Wol+	Wol−	*p*-value	Wol+	Wol−	*p*-value
*w*^Dah^	1.819	1.628	<0.05	62.76	50.65	<0.01
InRDN/+	1.829	1.683	<0.05	62.75	52.52	<0.01
daGAL4/+	1.83	1.661	<0.05	63.72	49.08	<0.01
daGAL4/InRDN	1.239	0.728	<0.05	27.7	1.98	<0.01

**Figure 2.**
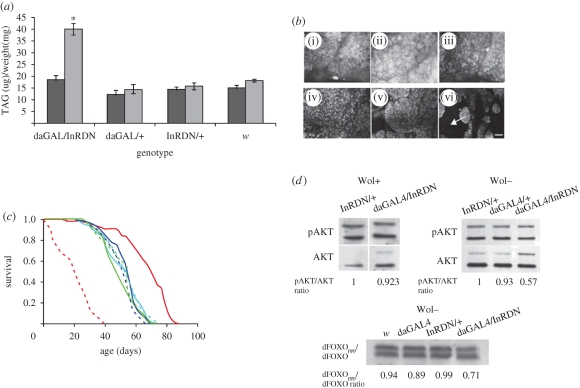
The effect of *Wolbachia* infection status on fat, survival and AKT phosphorylation. (*a*) TAG concentration of individual whole virgin females (*n* = 12). Wol+ *w*^Dah^ = 15.15 µg mg^−1^ of fly weight; Wol− *w*^Dah^ = 18.15 µg mg^−1^; Wol+ InRDN/+ = 14.38 µg mg^−1^; Wol− InRDN/+ = 15.74 µg mg^−1^; Wol+ daGAL4/dInRDN = 18.29 µg mg^−1^; Wol− daGAL4/dInRDN = 40.07 µg mg^−1^; Wol+ daGAL4/+ = 12.25 µg mg^−1^; and Wol− daGAL4/+ = 14.37 µg mg^−1^. Data were subjected to two-way ANOVA, with genotype (*p* < 0.0001) and *Wolbachia* status (*p* < 0.0001) having significant effects. There was a significant interaction effect of genotype and *Wolbachia* status (*p* < 0.0001). Planned comparisons of the mean TAG level by genotype/*Wolbachia* status were made using the Tukey HSD. Error bars are s.e.m. and asterisk indicates significant difference from all groups, *p* < 0.05. Black bars, *Wolbachia*-infected; grey bars, -uninfected. (*b*) Adult abdominal fat body morphology for each genotype: (i) Wol+ InRDN/+; (ii) Wol+ daGAL4/+; (iii) Wol+ daGAL4/InRDN; (iv) Wol− InRDN/+; (v) Wol− daGAL4/+; (vi) Wol− daGAL4/InRDN. Arrow indicates area of missing tissue and scale bar is 20 µm. (*c*) Survival of *Wolbachia*-infected and -uninfected daGAL4/InRDN once-mated females and controls. Median lifespans and statistical analyses are given in table 2. Red solid line, daGAL4/InRDN Wol+; red dashed line, daGAL4/InRDN Wol−; dark blue solid line, daGAL4 Wol+; dark blue dashed line, daGAL4 Wol−; green solid line, InRDN Wol+; green dashed line, InRDN Wol−; light blue solid line, *w* Wol+; light blue dashed line, *w* Wol−. (*d*) Levels of AKT and dFOXO phosphorylation were detected by Western blotting. For AKT phosphorylation, representative blots of two independent experiments each for Wol+ and Wol− backgrounds are shown. The upper two bands of each panel are phospho-AKT (pAKT) and the bottom two bands are tAKT. The presence of two bands is due to expression of two isoforms of AKT. pAKT/tAKT relative ratio was estimated from the signal intensity of the lower bands of both pAKT and AKT, which was averaged by ROI analysis in ImageJ. For dFOXO phosphorylation in the Wol− background, a single experiment was performed. The ratio of phosphorylated to unphosphorylated dFOXO (dFOXO_ppp_/dFOXO ratio) was estimated from the intensity of the upper (dFOXO_ppp_) to the lower (dFOXO) band in ImageJ.

Increases in TAG levels and alterations in adult fat tissue morphology are often associated with reduced IIS (Bohni *et al*. 1999; Tatar *et al*. 2001). Wol+ daGAL4/InRDN females had normal TAG levels (figure 2*a*) and adult fat body morphology (figure 2*b*) compared to Wol+ controls. In contrast, Wol− daGAL4/InRDN females had significantly higher TAG levels (figure 2*a*; Tukey HSD, *p* < 0.05) and alterations in fat body morphology (figure 2*b*) compared to all other genotypes. Unlike the morphologically normal abdominal fat tissue of *Wolbachia*-infected daGAL4/InRDN and control flies (figure 2*b*(i–iii)), this tissue was misshapen and shrunken in Wol− daGAL4/InRDN flies (figure 2*b*(vi)), similar to that seen under starvation conditions (Scott *et al*. 2004). For these IIS-related phenotypes, *Wolbachia* status had no effect on control genotypes (figure 2*a*,*b*).

Lifespan is another trait that responds to changes in IIS and the effect of reducing the activity of the pathway can be either positive or negative. Some mutations or genetic manipulations of IIS, such as strong, ubiquitous downregulations, are often detrimental to lifespan (Clancy *et al*. 2001; Tatar *et al*. 2001; Taguchi *et al*. 2007; Selman *et al*. 2008), but moderate or tissue-specific reductions in IIS can extend it (Clancy *et al*. 2001; Tatar *et al*. 2001; Giannakou *et al*. 2004; Hwangbo *et al*. 2004; Broughton *et al*. 2005; Selman *et al*. 2008). It appears that alterations of specific IIS components at particular times or in specific tissues may be important, or that lifespan may peak at an intermediate level of IIS activity. Interestingly, Wol+ daGAL4/UAS-InRDN once-mated females showed a large extension of median and maximum lifespan (figure 2*c*; table 2 for statistical analysis; electronic supplementary material, figure 2*a*), whereas Wol− daGAL4/InRDN females were very short-lived (figure 2*c*; table 2 for statistical analysis; electronic supplementary material, figure 2*b*). For control genotypes, *Wolbachia* infection status had no effect on lifespan (figure 2*c*; table 2 for statistical analysis; electronic supplementary material, figure 2*a*–*c*). This effect of *Wolbachia* removal to drastically shorten the lifespan of daGAL4/UAS-InRDN females is consistent with an accentuation of the inhibitory effect of InRDN expression, resulting in a reduction in IIS to levels detrimental to survival. The effect of over-expression of InRDN on lifespan of males in the Wol+ strain was also measured, and a slight reduction in median lifespan was found (electronic supplementary material, figure 2*d*). This detrimental effect of InRDN expression to male lifespan appeared to be accentuated in the absence of *Wolbachia* as only very few Wol− daGAL4/UAS-InRDN males enclosed, and those that did died within a few days. This sex difference in the effect on survival is typical for IIS mutants in *Drosophila* (Clancy *et al*. 2001; Tatar *et al*. 2001; Giannakou *et al*. 2004; Broughton *et al*. 2005) and in mice (Holzenberger *et al*. 2003; Selman *et al*. 2008).

**Table 2.** Median and maximum lifespans and statistical comparisons of survivorship data from figure 2*c*. Each pairwise comparison of survivorship data by genotype and *Wolbachia* infection status was made using the log-rank test, and χ^2^ *p*-values are given.

genotype/*Wolbachia* status	daGAL/InRDN+	daGAL/InRDN−	daGAL+	InRDN−	daGAL−	InRDN+	*w*−	*w*+
median lifespan (days)	70.5	21.4	54.0	54.0	49.0	45.5	54.0	54.0
maximum lifespan (days)	82	36.0	66.0	65.0	63.0	66.0	69.0	66.0
daGAL/InRDN+		2.197×10^−36^	1.632×10^−16^	3.861×10^−17^	1.853×10^−19^	3.707×10^−18^	2.923×10^−13^	1.488×10^−13^
daGAL/InRDN−			3.695×10^−35^	5.675×10^−29^	2.102×10^−32^	2.073×10^−31^	6.635×10^−27^	1.417E×10^−25^
daGAL+				0.2081	0.00976	0.08486	0.46632	0.75395
InRDN−					0.20105	0.52295	0.12628	0.20625
daGAL−						0.88930	0.01548	0.03060
InRDN+							0.06816	0.19695
*w*−								0.67115
*w*+								

The previous data suggested that *Wolbachia* acts to enhance IIS. As a further indicator of a change in the magnitude of IIS downregulation in daGAL4/UAS-InRDN flies owing to *Wolbachia* infection, the activation of two key downstream effectors of IIS—AKT and the fly forkhead box O transcription factor, dFOXO—were measured. The Ser505 of AKT is a target of the TOR/RICTOR complex, the phosphorylation of which is required for the full activation of the AKT protein (Sarbassov *et al*. 2005) and which is used as an indicator of AKT activity (Hietakangas & Cohen 2007). We measured the phosphorylation state of this serine using an antibody specific to the phosphoS505-AKT form of the protein (pAKT) and an antibody to total AKT (tAKT) (which recognizes both phosphorylated and unphosphorylated forms of AKT) in Wol+ and Wol− flies in Western blots. The level of AKT activation was then estimated by normalizing the pAKT signal to the tAKT signal. Only Wol− daGAL4/InRDN females showed a reduced pAKT/tAKT ratio compared with controls in two independent experiments (figure 2*d*), suggesting that the downregulation of IIS owing to daGAL4-driven expression InRDN was stronger in the absence of *Wolbachia* than in its presence. Active AKT phosphorylates dFOXO, and this phosphorylation retards the migration of dFOXO on SDS–PAGE (Puig *et al*. 2003). To confirm the reduction in AKT activity in Wol− daGAL4/InRDN females, we examined levels of phosphorylated dFOXO. Indeed, consistent with a reduction in AKT activity in the Wol− daGAL4/InRDN females, we observed a decrease in the ratio of slower-migrating/phosphorylated to faster-migrating/unphosphorylated dFOXO in these flies (figure 2*d*).

Interestingly, the enhanced IIS-related phenotypes of the Wol− daGAL4/InRDN females resembled those due to a strong downregulation of the pathway obtained using daGAL-driven expression of UAS-PTEN. The essential function of the phosphoinositide phosphatase PTEN is to antagonize the effects of PI3K by dephosphorylation of the second messenger phosphatidylinositol-3,4,5-trisphosphate (PIP_3_; Stocker *et al*. 2002). UAS-PTEN expression thus downregulates IIS by converting PIP_3_ to PIP_2_ (Goberdhan *et al*. 1999; Maehama *et al*. 2004). Even in the presence of *Wolbachia*, daGAL/UAS-PTEN flies showed severe IIS-related phenotypes including growth, fecundity, fat and AKT phosphorylation, and they were very short-lived (electronic supplementary material, figures 1–3).

Taken together, these data suggest that the removal of the endosymbiont *Wolbachia* from daGAL4/InRDN females caused a greater inhibitory effect of InRDN expression on IIS, resulting in enhanced IIS-related phenotypes. The similar but less pronounced reduction in weight and fecundity seen upon removal of *Wolbachia* from control genotypes suggests that these two phenotypes may be the most sensitive to changes in IIS and demonstrates that *Wolbachia*'s effect is not specific to the daGAL4/InRDN genotype.

### (b) Removal of *Wolbachia* enhances the effect of expression of InRDN in adult fat body

The quantitatively different effects of InRDN expression on IIS-related phenotypes in the presence and absence of *Wolbachia*, described above, suggested a dose dependency of IIS downregulation. To begin to determine if these effects are general, we used the RU486 GeneSwitch-inducible GAL/UAS system (Osterwalder *et al*. 2001; Roman *et al*. 2001) to express InRDN in adult fat body. Manipulation of IIS by over-expression of the key IIS effector forkhead transcription factor, dFOXO, in this tissue using the inducible S_1_106-GS driver has previously been shown to be sufficient to extend lifespan (Giannakou *et al*. 2004). This driver was initially reported as expressed in adult fat body (Roman *et al*. 2001) and has since been shown also to express in the digestive system and testes (Poirier *et al*. 2008).

**Figure 3.**
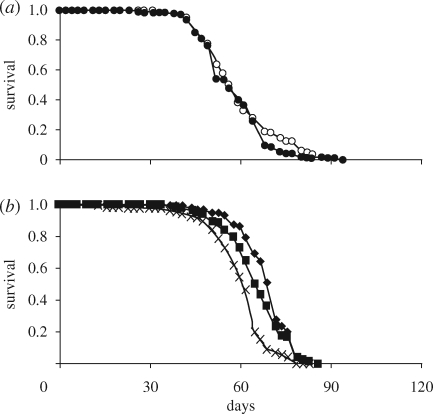
Expression of InRDN in the abdominal adult fat body extends lifespan only in the absence of *Wolbachia*. Lifespan experiments were done in (*a*) *Wolbachia*-infected and (*b*) *Wolbachia-*uninfected flies. (*a*) Survival of Wol+ S_1_,106-GS/InRDN females was not affected by administration of 200 µM of RU486 inducer. The median lifespan was 56 days (*n* = 185) in the absence of the drug and 56 days (*n* = 188) in the presence of the drug. Open circles, S106/InRDN RU−; filled circles, S106/InRDN RU+. (*b*) Survival of Wol-S_1_,106-GS/InRDN on 200 and 300 µM RU486 concentrations compared with uninduced genetically identical flies (0 µM RU486). Individual median lifespans were 61 days on 0 µM (*n* = 141), 65 days on 200 µM (*n* = 137, χ^2^ *p* = 1.16 × 10^−7^) and 69 days on 300 µM (*n* = 130, χ^2^ *p* = 3.65 × 10^−15^). Crosses, S106/InRDN RU−; filled squares, S106/InRDN 200; filled diamonds, S106/InRDN 300.

Expression of InRDN using the S_1_106-GS driver under the same induction conditions as in Giannakou *et al*. (2004) had no effect on lifespan in the presence of *Wolbachia* (figure 3*a*). However, removal of *Wolbachia* from S_1_106-GS/UAS-InRDN female flies resulted in extension of lifespan and revealed a dose-dependent effect of expression of InRDN (figure 3*b*). At 200 µM, RU486 inducer Wol− S_1_106-GS/UAS-InRDN females showed a 6.5 per cent increase in median lifespan (χ^2^ *p* = 1.16×10^−7^) and at 300 µM RU486 a 13 per cent increase (χ^2^ *p* = 3.65×10^−15^) compared with the same genotype at 0 µM RU486. There was no effect of RU486 on survival of driver control female flies (electronic supplementary material, figure 4). S_1_106-GS/InRDN expression had no effect on fecundity or lipid levels (electronic supplementary material, figure 5), confirming the previous study modulating IIS in this tissue (Giannakou *et al*. 2004, 2007). pAKT levels in the long-lived Wol− S_1_106-GS/InR-DN females were found to be indistinguishable from controls (electronic supplementary material, figure 5).

These data suggest that removal of *Wolbachia* from Wol+ normal-lived S_1_106-GS/InRDN females acted to increase the inhibitory effect of InRDN in this tissue to a threshold sufficient for lifespan extension. The data further suggest that ubiquitous expression of InRDN by daGAL4 may have affected fecundity, growth and fat phenotypes via expression during development and/or in other tissues.

## 5. Discussion

The data presented here demonstrate that mutant IIS and its consequent phenotypes in *Drosophila* can be enhanced by removal of *Wolbachia*, suggesting that one of the normal consequences of *Wolbachia* infection is enhanced IIS. The data are consistent with a general effect on IIS, and not a specific interaction with mutants such as the daGAL4/InRDN genotype, because removal of *Wolbachia* from control strains had similar, although weaker, effects. More work will be needed both to determine the generality of these kinds of effects and exactly how *Wolbachia* modifies IIS-related phenotypes.

Evolutionary considerations suggest that the mechanisms by which *Wolbachia* interacts with IIS may have its origin in the way that these endosymbionts are transmitted. Their habitat is inside the cytoplasm of cells, and they are transmitted maternally, via infection of eggs (Stouthamer *et al*. 1999). *Wolbachia* are thought to persist in host populations by providing females with a reproductive advantage via mechanisms that can include male killing, feminization of genetic males and cytoplasmic incompatibility (see Stouthamer *et al*. 1999 for review). In *D. melanogaster*, an effect of *Wolbachia* infection is to confer reproductive advantage through cytoplasmic incompatibility (Hoffmann *et al.* 1998). *Wolbachia*-infected females can successfully mate with infected or uninfected males, but uninfected females are incompatible with infected males. Thus, *Wolbachia* infection can spread and persist in the host population, although other mechanisms must also be involved (Olsen *et al*. 2001; Fry *et al*. 2004; Weeks *et al*. 2007; Brownlie *et al*. 2008).

In evolutionary terms, the interests of the female host and of the symbiont in host egg production are identical. Both host and symbiont are selected to maximize host fitness through this route. In populations of flies that are infected with *Wolbachia*, part of the response to selection for an optimal regime of host egg production through the life history may thus be attributable to evolution of the effects of the symbiont on host reproduction. However, this alone would not explain why *Wolbachia* would act as seen in this study, solely to promote *high* host fecundity. A possible explanation could come from events during adaptation to life in the laboratory. The life history of *Drosophila* evolves so as to optimize fitness under the prevailing culture regime (Sgro & Partridge 2000; Spencer & Promislow 2002). The usual regime consists of the transfer of young adults to fresh culture vials, with consequent selection for rapid maturation and copious early reproduction. This produces evolutionary increases in the speed of pre-adult development and in female fecundity relative both to the levels seen in wild flies and in laboratory stocks maintained with overlapping generations. These traits that are favoured in laboratory culture are also promoted by increased IIS, although it has not yet been determined whether laboratory adaptation is achieved in whole or in part by increased activity of this pathway. In stocks that are infected with *Wolbachia*, part of the adaptation to laboratory conditions may be achieved by the evolution of the symbiont so as to alter host physiology in ways that promote an optimal regime of egg production through copious, early female reproduction. One prediction of the hypothesis is that these effects of *Wolbachia* would be expected to be particularly obvious in strains that have undergone laboratory adaptation, in traits promoting fitness through the regime of fecundity and in females. Alternatively, interactions of IIS with the *Wolbachia* infection itself could play a role.

The tetracycline treatment used for the removal of *Wolbachia* has itself been found to result in a decline of mitochondrial activity and increase in mitochondrial DNA density, which may increase insulin insensitivity regardless of the removal of the endosymbionts (Ballard & Melvin 2007). However, this study measured the effects only two generations after treatment and, because the effect is based on damage to the mitochondria, it is expected to decline rapidly. The parental strains used in the current study were reared on normal food for at least five generations following tetracycline treatment to restore flora in their intestine prior to the generation of experimental flies for analysis. That such a regime does not produce ill-effects as a result of the tetracycline treatment itself is supported by the finding that tetracycline treatment of already-*Wolbachia*-free flies had no effect on lifespan when tested several generations after treatment (Toivonen *et al*. 2007).

The variability of the phenotypes owing to IIS reduction in the fly is probably due to the complex nature of the pathway itself, as well as the developmental stage and tissue affected by the genetic manipulation in question. However, the dose dependency of IIS downregulation identified here supports the idea that some of the variability could be related to differing strengths of IIS downregulation. Over-expression of the FOXO transcription factor in the fly eye imaginal disks induced cell death (Junger *et al*. 2003), whereas the same gene expressed in the adult fat body extended lifespan (Giannakou *et al*. 2004; Hwangbo *et al*. 2004). It is not known what intracellular differences exist between the adult fat body and imaginal disks that separate FOXO's longevity function from its apoptotic function, but a likely explanation could be differences in the level of FOXO activation in the two systems. Interestingly, a measurable reduction of AKT phosphorylation correlated with malformation of the adult fat body, sterility and shortened lifespan, which, given the role of AKT activity in preventing apoptosis, could suggest that the short lifespan of the *pkb*^*3*^ mutant was due to its reduced AKT activity level (Stocker *et al*. 2002).

Despite the uncertainties as to the mechanism by which IIS is modified, the *Wolbachia* effect has implications for studies of IIS in *Drosophila*. It is desirable to ensure that the *Wolbachia* status of all strains being compared is the same, to rule out any possibility that traits apparently attributable to altered IIS are in fact owing to differences in *Wolbachia* status. However, provided this stricture is observed, alteration in *Wolbachia* status could be a convenient way of altering the intensity of IIS. The initial state of IIS as well as the stage and tissue specificity of the IIS manipulation may determine how a change in *Wolbachia* status affects IIS phenotypes.

## 6. Conclusion

The data presented here show that the effects of mutant IIS are dose-dependent, and that *Wolbachia* acts to enhance IIS, thus modifying the extent to which IIS mutants affect signalling and the consequent pleiotropic phenotypes of the pathway. Furthermore, for some of these phenotypes, such as lifespan, the phenotype peaks at an intermediate level of IIS downregulation.

## References

[RSPB20090778C1] Ballard J. W. O. Melvin R. G. 2007 Tetracycline treatment influences mitochondrial metabolism and mtDNA density two generations after treatment in *Drosophila*. Insect Mol. Biol. 16, 799–802 18093008 10.1111/j.1365-2583.2007.00760.x

[RSPB20090778C2] Bass T. M. Grandison R. C. Wong R. Martinez P. Partridge L. Piper M. D. W. 2007 Optimization of dietary restriction protocols in *Drosophila*. J. Gerontol. 62, 1071–1081 10.1093/gerona/62.10.1071 PMC4335187 17921418

[RSPB20090778C3] Bohni R. Riesgo-Escovar J. Oldham S. Brogiolo W. Stocker H. Andruss B. F. Beckingham K. Hafen E. 1999 Autonomous control of cell and organ size by CHICO, a *Drosophila* homolog of vertebrate IRS1-4. Cell 97, 865–875 (doi:10.1016/S0092-8674(00)80799-0) 10399915 10.1016/s0092-8674(00)80799-0

[RSPB20090778C4] Bourtzis K. Nirgianaki A. Markakis G. Savakis C. 1996 *Wolbachia* infection and cytoplasm incompatibility in *Drosophila* species. Genetics 144, 1063–1073 8913750 10.1093/genetics/144.3.1063 PMC1207602

[RSPB20090778C5] Brogiolo W. Stocker H. Ikeya T. Rintelen F. Fernandez R. Hafen E. 2001 An evolutionarily conserved function of the *Drosophila* insulin receptor and insulin-like peptides in growth control. Curr. Biol. 11, 213–221 (doi:10.1016/S0960-9822(01)00068-9) 11250149 10.1016/s0960-9822(01)00068-9

[RSPB20090778C6] Broughton S. J. 2005 Longer lifespan, altered metabolism and stress resistance in *Drosophila* from ablation of cells making insulin-like ligands. Proc. Natl Acad. Sci. USA 102, 3105–3110 (doi:10.1073/pnas.0405775102) 15708981 10.1073/pnas.0405775102 PMC549445

[RSPB20090778C7] Brownlie J. C. Cass B. N. Riegler M. Witsenburg J. J. Iturbe-Ormaetxe I. McGraw E. A. O'Neill S. L. 2008 Evidence for metabolic provisioning by a common invertebrate endosymbiont, *Wolbachia pipientis*, during periods of nutritional stress. PLoS Pathog. 5, e1000368 (doi:10.1371/journal.ppat.1000368) 19343208 10.1371/journal.ppat.1000368 PMC2657209

[RSPB20090778C8] Butler A. A. Le Roith D. 2001 Control of growth by the somatropic axis: growth hormone and the insulin-like growth factors have related and independent roles. Annu. Rev. Physiol. 63, 141–164 (doi:10.1146/annurev.physiol.63.1.141) 11181952 10.1146/annurev.physiol.63.1.141

[RSPB20090778C9] Clancy D. J. Gems D. Harshman L. G. Oldham S. Stocker H. Hafen E. Leevers S. J. Partridge L. 2001 Extension of life-span by loss of CHICO, a *Drosophila* insulin receptor substrate protein. Science 292, 104–106 (doi:10.1126/science.1057991) 11292874 10.1126/science.1057991

[RSPB20090778C10] Clark M. E. Anderson C. L. Cande J. Karr T. L. 2005 Widespread prevalence of *Wolbachia* in laboratory stocks and the implications for *Drosophila* research. Genetics 170, 1667–1675 (doi:10.1534/genetics.104.038901) 15937134 10.1534/genetics.104.038901 PMC1449785

[RSPB20090778C11] Colberg S. 2007 Physical activity, insulin action, and diabetes prevention and control. Curr. Diabetes Rev. 3, 176–184 (doi:10.2174/157339907781368986) 18220669 10.2174/157339907781368986

[RSPB20090778C12] Del Aguila L. F. Krishnan R. K. Ulbrecht J. S. Farrell P. A. Correll P. H. Lang C. H. Zierath J. R. Kirwan J. P. 2000 Muscle damage impairs insulin stimulation of IRS-1, PI 3-kinase, and Akt-kinase in human skeletal muscle. Am. J. Physiol. Endocrinol. Metab. 279, E206–E212 10893341 10.1152/ajpendo.2000.279.1.E206

[RSPB20090778C13] Depke M. Fusch G. Domanska G. Geffers R. Völker U. Schuett C. Kiank C. 2008 Hypermetabolic syndrome as a consequence of repeated psychological stress in mice. Endocrinology 149, 2714–2723 (doi:10.1210/en.2008-0038) 18325986 10.1210/en.2008-0038

[RSPB20090778C14] Dionne M. S. Pham L. N. Shirazu-Hiza M. M. Schneider D. S. 2006 Akt and FOXO dysregulation contribute to infection-induced wasting in *Drosophila*. Curr. Biol. 16, 1977–1985 (doi:10.1016/j.cub.2006.08.052) 17055976 10.1016/j.cub.2006.08.052

[RSPB20090778C15] Fry A. J. Palmer M. R. Rand D. M. 2004 Variable fitness effects of *Wolbachia* infection in *Drosophila melanogaster*. Heredity 93, 379–389 (doi:10.1038/sj.hdy.6800514) 15305172 10.1038/sj.hdy.6800514

[RSPB20090778C16] Giannakou M. E. Goss M. Junger M. A. Hafen E. Leevers S. J. Partridge L. 2004 Long-lived *Drosophila* with overexpressed dFOXO in adult fat body. Science 305, 361 (doi:10.1126/science.1098219) 15192154 10.1126/science.1098219

[RSPB20090778C17] Giannakou M. E. Goss M. Jacobson J. Vinti G. Leevers S. J. Partridge L. 2007 Dynamics of the action of dFOXO on adult mortality in *Drosophila*. Aging Cell 6, 429–438 (doi:10.1111/j.1474-9726.2007.00290.x) 17465980 10.1111/j.1474-9726.2007.00290.x

[RSPB20090778C18] Goberdhan D. C. I. Paricio N. Goodman E. C. Mlodzik M. Wilson C. 1999 *Drosophila* tumor suppressor *PTEN* controls cell size and number by antagonizing the Chico/PI3-kinase signaling pathway. Genes Dev. 13, 3244–3258 (doi:10.1101/gad.13.24.3244) 10617573 10.1101/gad.13.24.3244 PMC317204

[RSPB20090778C19] Grönke S. Beller M. Fellert S. Ramakrishnan H. Jäckle H. Kühnlein R. P. 2003 Control of fat storage by a *Drosophila* PAT domain protein. Curr. Biol. 13, 603–606 (doi:10.1016/S0960-9822(03)00175-1) 12676093 10.1016/s0960-9822(03)00175-1

[RSPB20090778C20] Gutierrez E. Wiggins D. Fielding B. Gould A. P. 2007 Specialized hepatocyte-like cells regulate *Drosophila* lipid metabolism. Nature 445, 275–280 (doi:10.1038/nature05382) 17136098 10.1038/nature05382

[RSPB20090778C21] Hietakangas Cohen 2007 Re-evaluating AKT regulation: role of TOR complex 2 in tissue growth. Genes Dev. 21, 632–637 (doi:10.1101/gad.416307) 17369395 10.1101/gad.416307 PMC1820936

[RSPB20090778C22] Hoffmann A. A. Hercus M. Dagher H. 1998 Population dynamics of the *Wolbachia* infection causing cytoplasmic incompatibility in *Drosophila melanogaster*. Genetics 148, 221–231 9475734 10.1093/genetics/148.1.221 PMC1459765

[RSPB20090778C23] Holden P. R. Jones P. Brookfield J. F. 1993 Evidence for a *Wolbachia* symbiont in *Drosophila melanogaster*. Genet. Res. 62, 23–29 7691685 10.1017/s0016672300031529

[RSPB20090778C24] Holzenberger M. Dupont J. Ducos B. Leneuve P. Geloen A. Even P. C. Cervera P. Le Bouc Y. 2003 IGF-1 receptor regulates lifespan and resistance to oxidative stress in mice. Nature 421, 182–187 (doi:10.1038/nature01298) 12483226 10.1038/nature01298

[RSPB20090778C25] Hurst G. D. Johnson A. P. Schulenburg J. H. Fuyama Y. 2000 Male-killing *Wolbachia* in *Drosophila*: a temperature sensitive trait with a threshold bacterial density. Genetics 156, 699–709 11014817 10.1093/genetics/156.2.699 PMC1461301

[RSPB20090778C26] Hwangbo D. S. Gershman B. Tu M. P. Palmer M. Tatar M. 2004 *Drosophila* dFOXO controls lifespan and regulates insulin signalling in brain and fat body. Nature 429, 562–566 (doi:10.1038/nature02549) 15175753 10.1038/nature02549

[RSPB20090778C27] Ikeya T. Galic M. Belawat P. Nairz K. Hafen E. 2002 Nutrient-dependent expression of insulin-like peptides from neuroendocrine cells in the CNS contributes to growth regulation in *Drosophila*. Curr. Biol. 12, 1293–1300 (doi:10.1016/S0960-9822(02)01043-6) 12176357 10.1016/s0960-9822(02)01043-6

[RSPB20090778C28] Ikezu T. Okamoto T. Yonezawa K. Tompkins R. G. Martyn J. A. 1997 Analysis of thermal injury-induced insulin resistance in rodents. Implication of postreceptor mechanisms. J. Biol. Chem. 272, 25 289–25 295 (doi:10.1074/jbc.272.40.25289) 10.1074/jbc.272.40.25289 9312146

[RSPB20090778C29] Jia K. Chen D. Riddle D. L. 2004 The TOR pathway interacts with the insulin signaling pathway to regulate *C. elegans* larval development, metabolism and life span. Development 131, 3897–3906 (doi:10.1242/dev.01255) 15253933 10.1242/dev.01255

[RSPB20090778C30] Junger M. A. Rintelen F. Stocker H. Wasserman J. D. Vegh M. Radimerski T. Greenberg M. E. Hafen E. 2003 The *Drosophila* forkhead transcription factor FOXO mediates the reduction in cell number associated with reduced insulin signaling. J. Biol. 2, 20 (doi:10.1186/1475-4924-2-20) 12908874 10.1186/1475-4924-2-20 PMC333403

[RSPB20090778C31] Kapahi P. Zid B. M. Harper T. Koslover D. Sapin V. Benzer S. 2004 Regulation of lifespan in *Drosophila* by modulation of genes in the TOR signaling pathway. Curr. Biol. 14, 885–890 (doi:10.1016/j.cub.2004.03.059) 15186745 10.1016/j.cub.2004.03.059 PMC2754830

[RSPB20090778C32] Karpac J. Jasper H. 2009 Insulin and JNK: optimizing metabolic homeostasis and lifespan. Trends Endocrinol. Metab. 20, 100–106 (doi:10.1016/j.tem.2008.11.004) 19251431 10.1016/j.tem.2008.11.004 PMC3227503

[RSPB20090778C33] Kimura K. D. Tissenbaum H. A. Liu Y. Ruvkun G. 1997 daf-2, an insulin receptor-like gene that regulates longevity and diapause in *Caenorhabditis elegans*. Science 277, 942–946 (doi:10.1126/science.277.5328.942) 9252323 10.1126/science.277.5328.942

[RSPB20090778C34] Liang H. Masoro E. J. Nelson J. F. Strong R. McMahan C. A. Richardson A. 2003 Genetic mouse models of extended lifespan. Exp. Gerontol. 38, 1353–1364 (doi:10.1016/j.exger.2003.10.019) 14698816 10.1016/j.exger.2003.10.019

[RSPB20090778C35] Lithgow G. J. White T. M. Melov S. Johnson T. E. 1995 Thermotolerance and extended life-span conferred by single-gene mutations and induced by thermal stress. Proc. Natl Acad. Sci. USA 92, 7540–7544 (doi:10.1073/pnas.92.16.7540) 7638227 10.1073/pnas.92.16.7540 PMC41375

[RSPB20090778C36] Maehama T. Kosaka N. Okahara F. Takeuchi K. Umeda M. Dixon J. E. Kanaho Y. 2004 Suppression of a phosphatidylinositol-3-kinase signal by a specific spliced variant of *Drosophila* PTEN. FEBS Lett. 565, 43–47 (doi:10.1016/j.febslet.2004.03.074) 15135050 10.1016/j.febslet.2004.03.074

[RSPB20090778C37] Mair W. Goymer P. Pletcher S. D. Partridge L. 2003 Demography of dietary restriction and death in *Drosophila*. Science 301, 1731–1733 (doi:10.1126/science.1086016) 14500985 10.1126/science.1086016

[RSPB20090778C38] McGraw E. A. O'Neill S. L. 2004 *Wolbachia pipientis*: intracellular infection and pathogenesis in *Drosophila*. Curr. Opin. Microbiol. 7, 67–70 (doi:10.1016/j.mib.2003.12.003) 15036143 10.1016/j.mib.2003.12.003

[RSPB20090778C39] Min K.-T. Benzer S. 1997 *Wolbachia*, normally a symbiont of *Drosophila*, can be virulent, causing degeneration and early death. Proc. Natl Acad. Sci. USA 94, 10 792–10 796 (doi:10.1073/pnas.94.20.10792) 10.1073/pnas.94.20.10792 PMC23488 9380712

[RSPB20090778C40] Nelson D. W. Padgett R. W. 2003 Insulin worms its way into the spotlight. Genes Dev. 17, 813–818 (doi:10.1101/gad.1090203) 12670864 10.1101/gad.1090203

[RSPB20090778C41] Oh S. W. Mukhopadhyay A. Svrzikapa N. Jiang F. Davis R. J. Tissenbaum H. A. 2005 JNK regulates lifespan in *Caenorhabditis elegans* by modulating nuclear translocation of forkhead transcription factor/DAF-16. PNAS 102, 4494–4499 (doi:10.1073/pnas.0500749102) 15767565 10.1073/pnas.0500749102 PMC555525

[RSPB20090778C42] Olsen K. Reynolds K. T. Hoffman A. A. 2001 A field cage test of the effects of the endosymbiont Wolbachia on *Drosophila melanogaster*. Heredity 86, 731–737 (doi:10.1046/j.1365-2540.2001.00892.x) 11595053 10.1046/j.1365-2540.2001.00892.x

[RSPB20090778C43] Osterwalder T. Yoon K. S. White B. H. Keshishian H. 2001 A conditional tissue-specific transgene expression system using inducible GAL4. PNAS 98, 12 596–12 601 (doi:10.1073/pnas.221303298) 10.1073/pnas.221303298 PMC60099 11675495

[RSPB20090778C44] Partridge L. Gems D. 2002 Mechanisms of ageing: public or private? Nat. Rev. Genet. 3, 165–175 (doi:10.1038/nrg753) 11972154 10.1038/nrg753

[RSPB20090778C45] Piper M. D. Selman C. McElwee J. J. Partridge L. 2008 Separating cause from effect: how does insulin/IGF signalling control lifespan in worms, flies and mice? J. Intern. Med. 263, 179–191 18226095 10.1111/j.1365-2796.2007.01906.x

[RSPB20090778C46] Poirier L. Shane A. Zheng J. Seroude L. 2008 Characterization of the *Drosophila* gene-switch system in aging studies: a cautionary tale. Aging Cell 7, 758–770 (doi:10.1111/j.1474-9726.2008.00421.x) 18691185 10.1111/j.1474-9726.2008.00421.x

[RSPB20090778C47] Puig O. Marr M. T. Ruhf M. L. Tjian R. 2003 Control of cell number by *Drosophila* FOXO: downstream and feedback regulation of the insulin receptor pathway. Genes Dev. 17, 2006–2020 (doi:10.1101/gad.1098703) 12893776 10.1101/gad.1098703 PMC196255

[RSPB20090778C49] Roman G. Endo K. Zong L. Davis R. L. 2001 P[Switch], a system for spatial and temporal control of gene expression in *Drosophila melanogaster*. Proc. Natl Acad. Sci. USA 98, 12 602–12 607 (doi:10.1073/pnas.221303998) 10.1073/pnas.221303998 PMC60100 11675496

[RSPB20090778C48] Romao I. Roth J. 2008 Genetic and environmental interactions in obesity and type 2 diabetes. J. Am. Diet Assoc. 108 (Suppl. 41), S24–S28 (doi:10.1016/j.jada.2008.01.022) 18358250 10.1016/j.jada.2008.01.022

[RSPB20090778C50] Rulifson E. J. Kim S. K. Nusse R. 2002 Ablation of insulin-producing neurons in flies: growth and diabetic phenotypes. Science 296, 1118–1120 (doi:10.1126/science.1070058) 12004130 10.1126/science.1070058

[RSPB20090778C51] Saltiel A. R. Kahn C. R. 2001 Insulin signalling and the regulation of glucose and lipid metabolism. Nature 414, 799–806 (doi:10.1038/414799a) 11742412 10.1038/414799a

[RSPB20090778C52] Sarbassov D. D. Guertin D. A. Ali S. M. Sabatini D. M. 2005 Phosphorylation and regulation of Akt/PKB by the rictor-mTOR complex. Science 307, 1098–1101 (doi:10.1126/science.1106148) 15718470 10.1126/science.1106148

[RSPB20090778C74] Scott R. C. Schuldiner O. Neufeld T. P. 2004 Role and regulation of starvation-induced autophagy in the *Drosophila* fat body. Dev. Cell. 7, 167–178 (doi:10.1016/j.devcel.2004.07.009) 15296714 10.1016/j.devcel.2004.07.009

[RSPB20090778C53] Selman C. 2008 Evidence for lifespan extension and delayed age-related biomarkers in insulin receptor substrate 1 null mice. FASEB J. 22, 807–818 (doi:10.1096/fj.07-9261com) 17928362 10.1096/fj.07-9261com

[RSPB20090778C54] Sgro C. M. Partridge L. 2000 Evolutionary responses of the life history of wild caught *Drosophila melanogaster* to two standard methods of laboratory culture. Am. Nat. 156, 341–353 (doi:10.1086/303394)

[RSPB20090778C55] Skorokhod A. Gamulin V. Gundacker D. Kavsan V. Muller I. M. Muller W. E. 1999 Origin of insulin receptor-like tyrosine kinases in marine sponges. Biol. Bull. 197, 198–206 (doi:10.2307/1542615) 10573839 10.2307/1542615

[RSPB20090778C56] Sokal R. R. Rohlf F. J. 1998 Biometry New York, NY: W.H. Freeman

[RSPB20090778C57] Spencer C. C. Promislow D. E. 2002 Genes, culture, and aging flies—what the lab can and cannot tell us about natural genetic variation for senescence. Sci. Aging Knowl. Environ. 20, e6 10.1126/sageke.2002.20.pe6 14602988

[RSPB20090778C58] Starr D. J. Cline T. W. 2002 A host–parasite interaction rescues *Drosophila* oogenesis defects. Nature 418, 76–79 (doi:10.1038/nature00843) 12097909 10.1038/nature00843

[RSPB20090778C59] Stocker H. Andjelkovic M. Oldham S. Laffargue M. Wymann M. P. Hemmings B. A. Hafen E. 2002 Living with lethal PIP3 levels: viability of flies lacking PTEN restored by a PH domain mutation in Akt/PKB. Science 295, 2088–2091 (doi:10.1126/science.1068094) 11872800 10.1126/science.1068094

[RSPB20090778C60] Stouthamer R. Breeuwer J. A. Hurst G. D. 1999 *Wolbachia pipientis*: microbial manipulator of arthropod reproduction. Annu. Rev. Microbiol. 53, 71–102 (doi:10.1146/annurev.micro.53.1.71) 10547686 10.1146/annurev.micro.53.1.71

[RSPB20090778C61] Taguchi A. Wartschow L. M. White M. F. 2007 Brain IRS2 signaling coordinates life span and nutrient homeostasis. Science 317, 369–372 (doi:10.1126/science.1142179) 17641201 10.1126/science.1142179

[RSPB20090778C62] Tatar M. Kopelman A. Epstein D. Tu M.-P. Yin C.-M. Garofalo R. S. 2001 A mutant *Drosophila* insulin receptor homolog that extends life-span and impairs neuroendocrine function. Science 292, 107 (doi:10.1126/science.1057987) 11292875 10.1126/science.1057987

[RSPB20090778C63] Tatar M. Bartke A. Antebi A. 2003 The endocrine regulation of aging by insulin-like signals. Science 299, 1346–1351 (doi:10.1126/science.1081447) 12610294 10.1126/science.1081447

[RSPB20090778C64] Toivonen J. M. Walker G. A. Martinez-Diaz P. Bjedov I. Driege Y. Jacobs H. T. Gems D. Partridge L. 2007 No influence of Indy on lifespan in *Drosophila* after correction for genetic and cytoplasmic background effects. PLoS Genet. 3, e95 (doi:10.1371/journal.pgen.0030095) 17571923 10.1371/journal.pgen.0030095 PMC1892600

[RSPB20090778C65] Van den Berghe G. 2001 Intensive insulin therapy in critically ill patients. N. Engl. J. Med. 345, 1359–1367 (doi:10.1056/NEJMoa011300) 11794168 10.1056/NEJMoa011300

[RSPB20090778C66] Vellai T. Takacs-Vellai K. Zhang Y. Kovacs A. L. Orosz L. Müller F. 2003 Influence of TOR kinase on lifespan in *C. elegans*. Nature 426, 620 (doi:10.1038/426620a) 14668850 10.1038/426620a

[RSPB20090778C67] Veneti Z. Clark M. E. Zabalou S. Karr T. L. Savakis C. Bourtzis K. 2003 Cytoplasmic incompatibility and sperm cyst infection in different *Drosophila–Wolbachia* associations. Genetics. 164, 545–552 12807775 10.1093/genetics/164.2.545 PMC1462605

[RSPB20090778C68] Wang M. C. Bohmann D. Jasper H. 2005 JNK extends life span and limits growth by antagonizing cellular and organism-wide responses to insulin signaling. Cell 121, 115–125 (doi:10.1016/j.cell.2005.02.030) 15820683 10.1016/j.cell.2005.02.030

[RSPB20090778C69] Weeks A. R. Turelli M. Harcombe W. R. Reynolds K. T. Hoffman A. A. 2007 From parasite to mutualist: rapid evolution of *Wolbachia* in natural populations of *Drosophila*. PLoS Biol. 5, e114 (doi:10.1371/journal.pbio.0050114) 17439303 10.1371/journal.pbio.0050114 PMC1852586

[RSPB20090778C70] Williams K. D. Busto M. Suster M. L. So A. K.-C. Ben-Shahar Y. Leevers S. J. Sokolowski M. B. 2006 Natural variation in *Drosophila melanogaster* diapauses due to the insulin-regulated PI3-Kinase. Proc. Natl Acad. Sci. USA 103, 15 911–15 915 (doi:10.1073/pnas.0604592103) 17043223 10.1073/pnas.0604592103 PMC1635102

[RSPB20090778C71] Wu Q. Zhang Y. Xu J. Shen P. 2005 Regulation of hunger-driven behaviors by neural ribosomal S6 kinase in *Drosophila*. Proc. Natl Acad. Sci. USA 102, 13 289–13 294 (doi:10.1073/pnas.0501914102) 10.1073/pnas.0501914102 PMC1201572 16150727

[RSPB20090778C72] Wullschleger S. Loewith R. Hall M. N. 2006 TOR signaling in growth and metabolism. Cell 124, 471–484 (doi:10.1016/j.cell.2006.01.016) 16469695 10.1016/j.cell.2006.01.016

[RSPB20090778C73] Zhou W. Rousset F. O'Neil S. 1998 Phylogeny and PCR-based classification of *Wolbachia* strains using *wsp* gene sequences. Proc. Biol. Sci. 265, 509–515 (doi:10.1098/rspb.1998.0324) 9569669 10.1098/rspb.1998.0324 PMC1688917

